# Fasting stress hyperglycemia ratio as a predictor of intramyocardial hemorrhage and adverse outcomes in ST-segment elevation myocardial infarction

**DOI:** 10.3389/fendo.2026.1761471

**Published:** 2026-02-11

**Authors:** Hang Zhao, Zhengyu Tao, Xu Wang, Xinli Li, Hao Chang, Ding-Kun Ji, Weiliang Xia, Meng Jiang, Jun Pu

**Affiliations:** 1 Department of Cardiology, Renji Hospital, State Key Laboratory of Systems Medicine for Cancer, School of Medicine, Shanghai Jiao Tong University, Shanghai, China; 2 State Key Laboratory for Innovation and Transformation of Luobing Theory, Department of Cardiology, The First Affiliated Hospital of Nanjing Medical University, Jiangsu Province Hospital, Nanjing, Jiangsu, China; 3 Hangzhou Institute of Medicine (HIM), Chinese Academy of Sciences, Hangzhou, Zhejiang, China; 4 Institute of Molecular Medicine (IMM), Shanghai Key Laboratory for Nucleic Acid Chemistry and Nanomedicine, Renji Hospital, School of Medicine, Shanghai Jiao Tong University, Shanghai, China; 5 Center for Aging and Cancer Research, Global Institute of Future Technology; State Key Laboratory of Systems Medicine for Cancer, Renji Hospital, School of Medicine and School of Biomedical Engineering, Shanghai Jiao Tong University, Shanghai, China

**Keywords:** cardiac magnetic resonance, intramyocardial hemorrhage, major adverse cardiac events, microvascular injury, stress hyperglycemia ratio, ST-segment elevation myocardial infarction

## Abstract

**Introduction:**

Intramyocardial hemorrhage (IMH) represents the most severe form of microvascular injury and is associated with poor prognosis in patients with ST-segment elevation myocardial infarction (STEMI) treated with primary percutaneous coronary intervention (PPCI). However, the associations of glycemic parameters with the occurrence of IMH remain unclear. We aimed to evaluate the association of fasting stress hyperglycemia ratio (SHR)—a novel metric that adjusts acute glucose for chronic glycemia—with the presence of IMH and to explore its relationship with clinical outcomes in patients with STEMI.

**Methods:**

This study utilized data from the prospective, multicenter EARLY-MYO-CMR registry (NCT03768453). We enrolled consecutive STEMI patients undergoing PPCI who had cardiac magnetic resonance (CMR) imaging within a week after the index infarction. The primary endpoint was the presence of IMH defined by CMR T2* mapping. A secondary clinical endpoint was the composite of major adverse cardiac events (MACE) during follow-up.

**Results:**

Among the 496 patients included in this study, 205 (41.3%) exhibited IMH. Multivariable analysis identified fasting SHR as the strongest independent predictor of IMH (adjusted odds ratio [aOR] per 0.1-unit increase: 1.21; 95% CI: 1.10–1.33, P<0.001), outperforming fasting blood glucose and HbA1c. This association was consistent in both non-diabetic (aOR: 1.27; P=0.001) and diabetic patients (aOR: 1.21; P=0.015). Restricted cubic spline analysis revealed a significant nonlinear relationship (P for nonlinearity=0.004), characterized by a rapid increase in IMH risk at lower SHR levels, where the study population was primarily concentrated, and remained consistently high thereafter. During a median follow-up of 25 months, elevated fasting SHR was significantly associated with an increased risk of MACE (Unadjusted hazard ratio per 0.1-unit increase: 1.20; 95% CI: 1.11–1.31; P<0.001; adjusted hazard ratio per 0.1-unit increase:1.20; 95% CI: 1.09–1.31; P<0.001), with Kaplan-Meier analysis confirming a significantly higher cumulative incidence of MACE in the high SHR group (log-rank P<0.001).

**Discussion:**

Fasting SHR was a potent, independent predictor of IMH in reperfused STEMI. Notably, the IMH risk escalates rapidly even at lower SHR levels, underscoring the critical need for early management of stress hyperglycemia. Elevated SHR was significantly associated with increased risk of MACE. These findings establish fasting SHR not only as a biomarker for microvascular injury but also as a pivotal tool for early risk stratification in STEMI.

## Graphical Abstract

**Figure:**
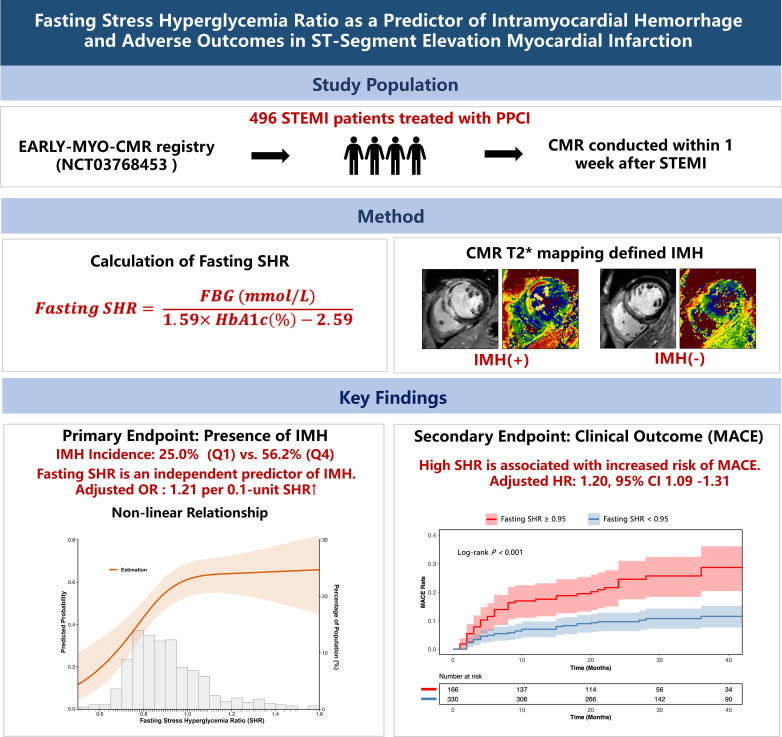
CMR, cardiac magnetic resonance; FBG, fasting blood glucose; IMH, intramyocardial hemorrhage; MACE, major adverse cardiac events; PPCI, percutaneous coronary intervention; SHR, stress hyperglycemia ratio; STEMI, ST-segment elevation myocardial infarction.

## 1 Introduction

Acute ST-segment elevation myocardial infarction (STEMI) remains a leading cause of cardiovascular mortality worldwide, with timely reperfusion therapy serving as the cornerstone of management (1). Despite successful epicardial coronary revascularization through primary percutaneous coronary intervention (PPCI), up to 50% of patients develop microvascular injury, a phenomenon encompassing both microvascular obstruction (MVO) and intramyocardial hemorrhage (IMH) (2). IMH represents the most severe form of ischemia-reperfusion injury, characterized by ultrastructural capillary damage and extravasation of erythrocytes into the myocardial interstitium. According to the Canadian Cardiovascular Society stages of acute reperfused myocardial infarction, patients who develop IMH following PPCI are at the highest risk for long-term major adverse cardiovascular events (3, 4). Identifying individuals at high risk for IMH could improve risk stratification and facilitate potential therapeutic targets to mitigate microvascular injury in acute coronary syndromes.

Cardiac magnetic resonance (CMR) imaging has emerged as the gold standard for characterizing microvascular complications, with T2*-based sequences specifically identifying IMH as hypointense regions within the infarct zone (5, 6).Current pathophysiological theories suggest IMH results from the combined effects of mechanical disruption of microvascular integrity during ischemia and reperfusion-induced inflammatory cascades (7). Previous studies have indicated that several high-risk features—such as greater infarct size, poorer left ventricular ejection fraction (LVEF), anterior infarct location, elevated inflammatory markers, and the use of glycoprotein IIb/IIIa inhibitors—may be associated with the development of IMH (7–9). While these factors highlight the importance of ischemic burden and inflammation, the association between dysglycemia and IMH remains unclear.​ Particularly, previous studies failed to establish a consistent association between conventional glycemic parameters (such as fasting blood glucose [FBG] and glycated hemoglobin [HbA1c])and the occurrence of IMH.

Stress hyperglycemia, a well-documented response to acute illness, has recently gained attention due to its potential association with adverse outcomes in patients with acute myocardial infarction (10). The stress hyperglycemia ratio (SHR) calculated as the ratio of admission glucose to chronic glycemic levels was developed to quantify acute dysmetabolism independent of pre-existing diabetes (11). More recently, fasting SHR—derived from fasting plasma glucose and ​​HbA1c—has emerged as a refined metric that minimizes confounding by prandial status and more accurately captures true stress-induced hyperglycemia (12). Previous studies have shown that fasting SHR is associated with left ventricular dysfunction and MVO in STEMI (13). However, the relationship between fasting SHR and the risk of IMH, as well as its association with long-term clinical outcomes, remains unexplored. Based on the pathophysiological interplay among hyperglycemia, inflammation, and microvascular integrity, we hypothesize that fasting SHR is independently associated with both the occurrence of IMH and subsequent adverse clinical outcomes in patients with reperfused STEMI.

This study aims to evaluate the association between fasting SHR and CMR-defined IMH, and to explore its prognostic value for major adverse cardiac events (MACE) in STEMI patients treated with PPCI. Our findings may provide novel insights into the metabolic factors associated with reperfusion injury and subsequent cardiovascular risk and thereby aiding the development of personalized management strategies for high-risk STEMI patients.

## 2 Materials and methods

### 2.1 Study design, clinical assessments, and endpoint definitions

Data for this study were obtained from the EARLY-MYO-CMR (EARLY assessment of MYOcardial tissue characteristics by CMR in STEMI, NCT03768453) registry, which was a prospective, multicenter registry recruiting consecutive STEMI patients who underwent CMR across 10 sites (14–16). The registry received approval from the institutional review board at each participating center and was conducted in accordance with the principles of the Declaration of Helsinki (2013). All participants provided written informed consent. Criteria for participant eligibility and exclusion were described in our previous studies (14–16). Briefly, patients were eligible for the registry if they 1) had a STEMI (as diagnosed by typical ischemic syndrome and electrocardiography manifestation of ST elevation in at least two contiguous precordial ≥2mm or peripheral leads ≥1mm) at an age over 18-year-old; 2) CMR examination performed within one week after symptom onset; and 3) provision of written informed consent. The key exclusion criteria of the EARLY-MYO-CMR registry were: 1) inability to adhere to the follow-up schedule; 2) any medical condition deemed by investigators to preclude study participation; or 3) a life expectancy of less than 6 months due to any condition.

For the present analysis, we included consecutive patients enrolled in the EARLY-MYO-CMR registry between January 2018 and December 2023. We further excluded patients with the following conditions: 1) those with poor - quality CMR images or missing essential CMR sequences; 2) patients with confirmed cardiomyopathy, congenital heart disease, severe arrhythmia, or significant valvular disease; and 3) patients without HbA1c measurement or FBG values within 24 hours after PPCI.

Diabetes was diagnosed if a history of diabetes was reported in the medical record, if the patient had an HbA1c of ≥ 6.5% on admission, or if the patient was currently receiving antidiabetic medication. FBG was measured from the blood sample obtained on the first morning after PPCI following at least 8 hours of overnight fasting. All samples were obtained prior to the administration of the first morning dose of insulin or any other glucose-lowering medications. The fasting SHR was calculated by dividing FBG (in mmol/L) by the estimated average glucose (eAG), derived from HbA1c using the formula: eAG (mmol/L) = 1.59 × HbA1c (%) − 2.59. Clinical characteristics, medical history, serum biochemical parameters, angiographic findings, and medication data were prospectively collected and entered into the database (14–17).

The primary outcome was the presence of IMH defined by CMR T2* mapping. Secondary endpoint was the composite of MACE, which encompassed all-cause death, nonfatal reinfarction, heart failure and stroke. The detailed definitions for MACE components have been published in our previous work (14–17). Briefly, all-cause death was defined as death of any cause and was considered cardiac unless a definite noncardiac cause can be established. Reinfarction was defined as recurrent chest pain associated with re-elevation of the ST segments in association with either re-elevation of the cardiac enzymes that met ACC/ESC committee criteria (18). Heart failure was defined as new-onset or worsening signs and symptoms of heart failure requiring urgent therapy (e.g., diuretics) and hospitalization during follow-up. Stroke was defined as either ischemic or hemorrhagic stroke showing a new focal neurologic deficit thought to be vascular in origin, with signs or symptoms lasting more than 24 hours. Clinical outcomes were assessed by clinical event committees based on trial specific definitions. When a patient experienced more than one event, the first event was counted. Censoring was applied for patients who remained event-free by the end of the study period (December 31, 2024) or were lost to follow-up prior to this date.

### 2.2 Coronary angiography and primary angioplasty

All procedures were performed according to standard techniques (13–16). All patients received 300 mg of aspirin and a loading dose of adenosine diphosphate receptor antagonists (300–600 mg clopidogrel or 180 mg ticagrelor) in the emergency room. During primary PCI, unfractionated heparin was administered to maintain an activated clotting time of approximately 300–350 seconds. When technically feasible, stenting of the infarct-related artery was performed, and the use of drug-eluting stents was encouraged. Glycoprotein IIb/IIIa inhibitors were not routinely given prior to the procedure; their intra- or post-procedural use was left to the treating interventional cardiologist’s judgment. The choice to perform thrombus aspiration was also at the discretion of the operator. Patients received guideline-directed therapy after intervention, including beta-blockers, angiotensin-converting enzyme inhibitors, or an angiotensin II receptor blocker, statins, and appropriate antithrombotic medications (19, 20).

### 2.3 CMR examination and analysis

The design of the CMR protocol was previously published (13, 14). Patients underwent CMR imaging within 7 days after PPCI, using a 1.5-T or 3-T systems (Achieva TX; Philips Healthcare, Best, the Netherlands). Cine imaging in multiplanar short-axis (TR/TE 3.2/1.6 ms, 30 phases, voxel size 2.0 × 1.6 × 8.0 mm^3^) and long-axis views (TR/TE 3.2/1.6 ms, 30 phases, voxel size 2.0 × 1.6 × 8.0 mm^3^) were obtained using a balanced steady-state free precession (SSFP) sequence. Intramyocardial hemorrhage assessment was performed using T2* mapping (TR/TE = 16/1.25+n*10 ms, flip angle = 20°, bandwidth = 1430 Hz/pixel, spatial resolution = 1.8 × 1.8 × 8.0 mm³). Infarct size and microvascular obstruction were determined using late gadolinium-enhancement (LGE) sequence (TR/TE 3.5/1.7 ms, temporal resolution 190 ms, voxel size 1.5 × 1.7 × 10 mm3 interpolated into 0.74 × 0.74 × 5 mm^3^) with a properly selected inversion time 10 minutes after administration of 0.2 mmol/kg Magnevist (Bayer Healthcare Pharmaceuticals Inc., Germany). All images were acquired in breath-hold. Default field of view was set at 350×350 mm^2^.

**Figure 1:**
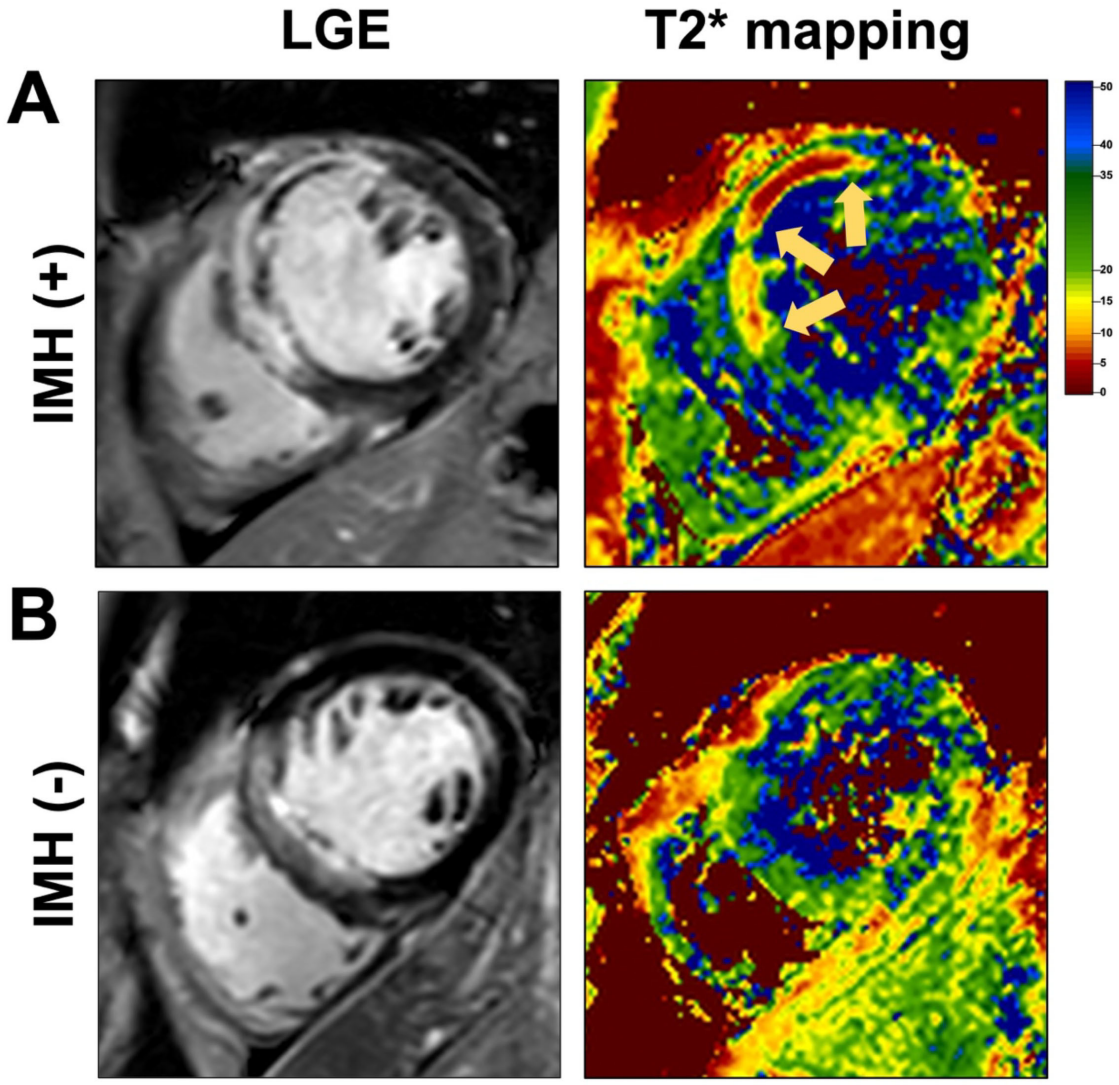
Representative cardiac magnetic resonance (CMR) images of **(A)** a patient with intramyocardial hemorrhage (IMH) and **(B)** a patient without IMH. For each case, the left panel shows the late gadolinium enhancement (LGE) image, and the right panel displays the corresponding T2* mapping image for IMH evaluation. IMH is visualized as a hypointense core within the hyperintense infarcted region (indicated by yellow arrows) on T2* mapping cardiovascular magnetic resonance images.

All CMR data were stored digitally in digital imaging and communications in medicine format for subsequent offline analysis. Images were analyzed at an independent core laboratory by two experts with>5 years of experience in cardiovascular imaging diagnosis and blinded to all clinical data using commercial software (CVI42 commercial, 5.2.0, Circle, Canada). Briefly, borders of epicardium, endocardium, infarcted myocardium, myocardial edema, IMH, and MVO were defined by the software with necessary manual corrections. Ventricular volumes and LVEF were calculated based on short-axis slices of cine images covering the whole heart. The presence of infarction was established on the basis of abnormalities in cine wall motion, rest first-pass myocardial perfusion, and delayed-enhancement imaging. The myocardial mass of late gadolinium (grams) was quantified using computer-assisted planimetry, and the territory of infarction was defined as myocardium with a signal intensity threshold of ≥5 standard deviations (SDs) of the nulled remote myocardium on LGE images. Infarct size was calculated as percentage of left ventricular mass (%LV). Any hypo-intensified area encircled by infarcted myocardium was defined as MVO. IMH refers to the hypointense core (T2* value <20 ms) within the region of myocardial infarction on T2* mapping (**Figure 1**).

### 2.4 Statistical analysis

Missing data were handled as follows: Patients lacking essential baseline measurements for FBG or HbA1c within 24 hours of PPCI were excluded from the final analysis cohort to ensure the calculation of fasting SHR. For other variables included in the analyses, the proportion of missing data was minimal (1.0% for lipid profiles). Therefore, a complete case analysis was performed, and no imputation methods were applied. Distribution of data was tested using the Shapiro-Wilk test. Continuous variables are presented as mean± SD for normally distributed data, or as median with 25th-75th percentiles for non-normally distributed data. Categorical variables are expressed as absolute numbers with corresponding percentages. Differences in baseline and CMR characteristics between patients with and without IMH were evaluated using the chi-square test for categorical variables and independent t-tests or Mann-Whitney U tests for continuous variables. Patients were further stratified into SHR quartiles (Q1-Q4). Inter-group differences were evaluated using one-way ANOVA (or the Kruskal-Wallis test) or the chi-square test, followed by Bonferroni-corrected *post-hoc* pairwise comparisons.

Univariable and multivariable logistic regression analyses were used to identify significant and independent predictors of IMH. Variables showing significant association (*P* < 0.10) with IMH in univariable were included into the multivariable model. Collinearity diagnostics were performed before modeling. Two primary models were constructed: Model 1 featured fasting SHR as the sole glycemic parameter (analyzed continuously as odds ratio [OR] per 0.1-unit increase), while Model 2 included only fasting blood glucose after excluding SHR (due to collinearity) and HbA1c (nonsignificant in univariate analysis). To assess whether the association between fasting SHR and IMH was modified by diabetes status, a multiplicative interaction term (fasting SHR × diabetes status) was introduced into the primary Model 1. Diabetes status and fasting SHR were forced into this specific interaction model as main effects. Following this interaction test, sensitivity analyses included stratified models by diabetes status. To rigorously evaluate the robustness of the association between SHR and IMH, a hierarchical regression approach comprising four progressive models was constructed. Hierarchical Model 1 adjusted for demographic and baseline risk factors, specifically sex and hypercholesterolemia. Model 2 extended Model 1 by additionally adjusting for angiographic and procedural characteristics, including infarct location (anterior infarct), pre-procedural Thrombolysis in Myocardial Infarction (TIMI) flow grade, and glycoprotein IIb/IIIa inhibitor use. Model 3 further incorporated the myocardial injury biomarker, peak cardiac troponin I. Finally, Model 4 served as the fully adjusted model, which additionally included CMR-derived functional and structural parameters: infarct size, LVEF, and MVO. Furthermore, causal mediation analysis was performed to investigate whether the effect of SHR on IMH was mediated by infarct size or the presence of MVO using the R ‘mediation’ package. We utilized a nonparametric bootstrap method with 1000 simulations to estimate the average causal mediation effect (ACME), average direct effect (ADE), and the proportion of the effect mediated, adjusting for potential confounders including age, gender, body mass index, the history of diabetes, and the use of glycoprotein IIb/IIIa inhibitors.

Restricted cubic spline (RCS) analysis within a multivariable logistic regression framework was performed to examine the potential nonlinear dose-response relationship between fasting SHR and the predicted probability of IMH. SHR was modeled as a continuous variable using a 3-knot RCS function, with knots placed at the 10th, 50th, and 90th percentiles of the SHR distribution. Frequency histograms and rug plots were superimposed on the RCS curves to visualize the data distribution. The multivariable model was adjusted for age, sex, and variables demonstrating a statistically significant association with IMH (*P* < 0.05) in prior analyses. Nonlinearity was assessed using a likelihood ratio test. The fitted spline curve and its 95% confidence interval (CI) were graphically presented.

The association between fasting SHR and MACE was evaluated using Cox proportional hazards regression models. SHR was analyzed as a continuous variable, and hazard ratios (HRs) with 95% CIs were calculated per 0.1-unit increment in SHR. Multivariable models were adjusted for clinically relevant covariates, including age, sex, hypertension, diabetes mellitus, Killip class, and anterior infarction location. To further explore the impact of SHR, exploratory analyses were conducted to estimate the HRs for individual components of the composite endpoint, including all-cause death, heart failure, myocardial reinfarction, and stroke. To assess the incremental prognostic value of SHR, we compared the predictive performance of a baseline model (comprising the aforementioned clinical covariates, including age, sex, hypertension, diabetes mellitus, Killip class, and anterior infarction location) with a full model (baseline model plus fasting SHR). Model discrimination was evaluated using Harrell’s C-index, and the statistical significance of the difference in C-indices between models was compared using the Likelihood Ratio Test. For graphical illustration of survival probabilities, an optimal cutoff value for fasting SHR was determined using maximally selected rank statistics (via the surv_cutpoint function in the survminer R package). Patients were stratified into two groups (high vs. low SHR) using this optimal cutoff. Cumulative incidence of MACE was estimated using the Kaplan–Meier method, and differences between groups were compared using the log-rank test. Patients lost to follow-up were treated as right-censored data. To address potential bias from loss to follow-up, a worst-case scenario sensitivity analysis (21) was conducted under the extreme assumption that all dropouts in the high SHR group remained event-free, whereas all dropouts in the low SHR group experienced MACE during the follow-up period.

*P* values<0.05 were considered statistically significant. When performing multiple comparisons among the SHR quartiles, statistical differences were determined at *P* < 0.0083. All analyses were performed using SPSS Statistics version 23 (IBM Corp) and R (version 4.3.1, R Foundation for Statistical Computing).

## 3 Results

### 3.1 Baseline characteristics

**Figure 2:**
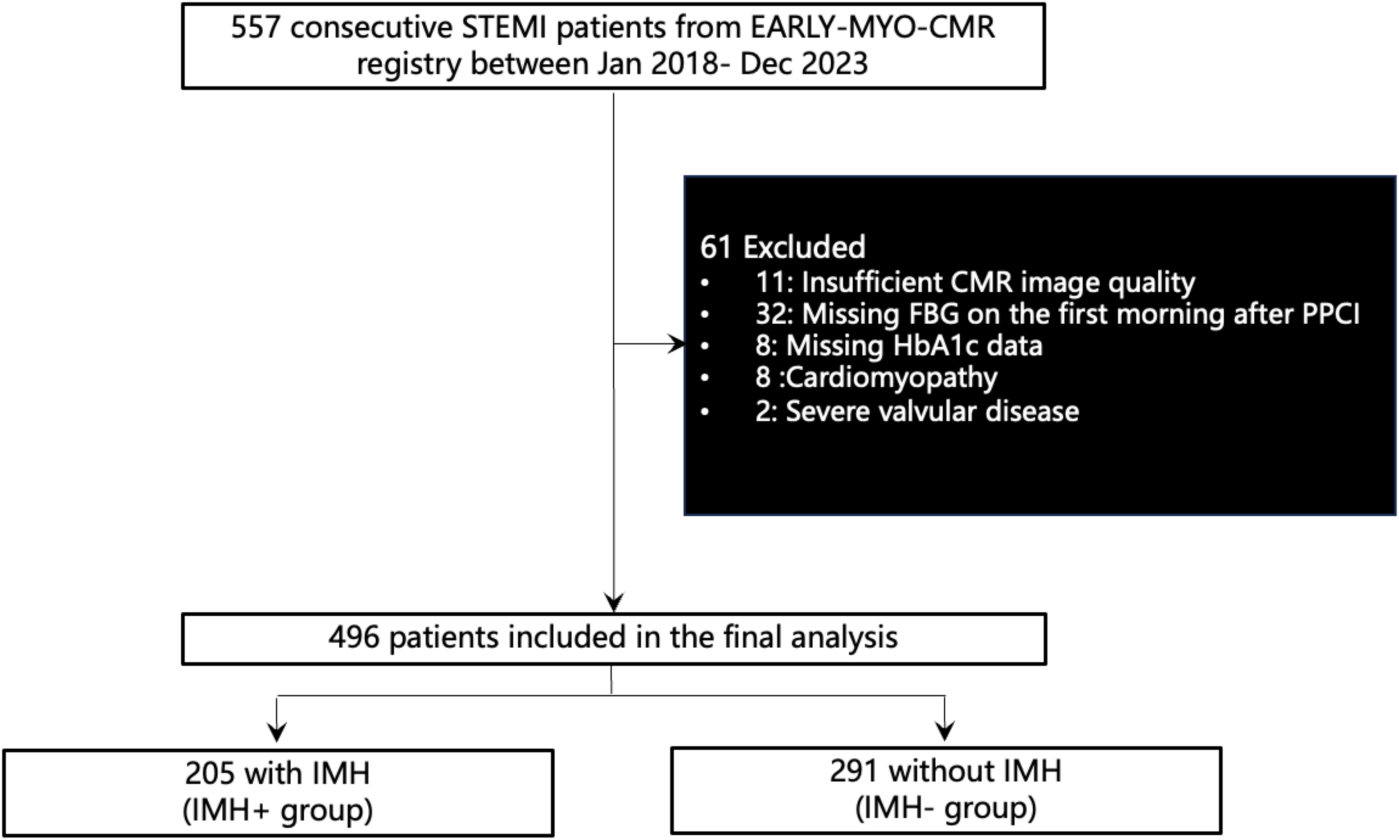
Study flowchart. CMR, cardiac magnetic resonance; FBG, fasting blood glucose; HbA1c, glycated hemoglobin A1c; IMH, Intramyocardial hemorrhage; PPCI, primary percutaneous coronary intervention; STEMI, ST-segment elevation myocardial infarction.

Between January 2018 and December 2023, a total of 557 patients were enrolled in the EARLY-MYO-CMR registry, and 496 patients were included in the final analysis according to the exclusion criteria of current study (**Figure 2**). Based on CMR findings, 205 patients (41.3%) were identified as having IMH. The baseline characteristics of patients with and without IMH are presented in **Table 1**.

**Table 1:** Baseline characteristics of the study population.

Variable	IMH+ (n=205)	IMH− (n=291)	*P* value
Demographic and clinical characteristics			
Age, years	58.33 ± 9.53	58.48 ± 10.52	0.869
Male sex, n (%)	185 (90.2%)	248 (85.2%)	0.098
Body mass index, kg/m²	25.26 ± 3.19	24.90 ± 3.11	0.211
SBP, mmHg	127.61 ± 20.54	128.96 ± 21.60	0.485
DBP, mmHg	78.95 ± 13.41	78.42 ± 13.01	0.659
Medical history			
Hypertension, n (%)	118 (57.6)	157 (54.0)	0.426
Diabetes, n (%)	57 (27.8)	81 (27.8)	0.994
Hyperlipidemia, n (%)	91 (44.4)	98 (33.7)	0.016
Current smoking, n (%)	114 (55.6)	158 (54.3)	0.772
Laboratory tests			
Fasting blood glucose, mmol/L	7.23 ± 2.89	6.76 ± 2.70	0.062
HbA1c, %	6.27 ± 1.43	6.40 ± 1.44	0.359
Fasting SHR	0.94 (0.84–1.07)	0.84 (0.77–0.96)	<0.001
Triglycerides, mmol/L	1.52 (1.11–2.39)	1.60 (1.18–2.46)	0.862
HDL cholesterol, mmol/L	1.03 (0.87–1.20)	1.03 (0.87–1.21)	0.708
LDL cholesterol, mmol/L	3.13 (2.55–3.67)	3.11 (2.44–3.71)	0.541
Creatinine, μmol/L	74.00 (62.95–86.00)	72.60 (60.00–84.00)	0.534
Peak cTnI, ng/mL	28.65 (12.95–59.83)	23.60 (8.07–43.81)	0.001
Infarct characteristics			
Anterior infarct, n (%)	134 (65.4)	166 (57.0%	0.062
Killip class ≥2, n (%)	8 (3.9)	9 (3.1)	0.414
Preinterventional TIMI flow grade, n (%)			0.022
0	170 (82.9)	227 (78.0)	
1	28 (13.7)	34 (11.7)	
2	7 (3.4)	23 (7.9)	
3	0 (0.0)	7 (2.4)	
Postinterventional TIMI flow grade, n (%)			0.451
3	194 (94.6)	279 (95.9)	
2	10 (4.9)	12 (4.1)	
1	0 (0.0)	0 (0.0)	
0	1 (0.5)	0 (0.0)	
Onset to reperfusion, min	275 (195–407)	293 (204–381)	0.892
Medications			
Aspirin	199 (97.1)	287 (98.6)	0.226
P2Y12 receptor inhibitor			0.422
Clopidogrel	166 (81.0)	227 (78.0)	
Ticagrelor	39 (19.0)	64 (22.0)	
GP IIb/IIIa Inhibitors	179 (87.3)	218 (74.9)	**0.001**
Beta-blockers	176 (85.9)	246 (84.5)	0.685
ACE Inhibitors	136 (66.3)	202 (69.4)	0.469
CMR Parameters			
Onset to CMR, d	5 (3-6)	4 (3-6)	0.732
Infarct size, % LV	26.67 ± 10.76	21.64 ± 10.35	<0.001
LVEF, %	46.27 ± 11.29	50.05 ± 10.97	<0.001
MVO present, n (%)	141 (68.8)	155 (53.3)	0.001

Values are mean ± SD, n (%), or median (interquartile range). Bold indicates *P* < 0.05.

ACE, angiotensin-converting enzyme; CMR, cardiac magnetic resonance; cTnI, cardiac troponin I; DBP, diastolic blood pressure; GP, glycoprotein; HbA1c, hemoglobin A1c; HDL, high-density lipoprotein; IMH, intramyocardial hemorrhage; LDL, low-density lipoprotein; LV, left ventricle; LVEF, left ventricular ejection fraction; MVO, microvascular obstruction; SBP, systolic blood pressure; SHR, stress hyperglycemia ratio; TIMI, Thrombolysis in Myocardial Infarction.

There were no significant differences in age, sex, and body mass index between the IMH - and IMH + groups (all *P* > 0.05). The fasting SHR was significantly elevated in the IMH+ group compared with the IMH- group (0.94 [0.84 - 1.07] vs.0.84 [0.77 - 0.96], *P* < 0.001), while FBG and HbA1c levels did not differ significantly between groups. The prevalence of hyperlipidemia was higher in the IMH + group (44.4% vs 33.7%, *P* = 0.016), while the prevalence of hypertension, diabetes, and smoking status was similar. Patients presenting IMH exhibited a higher prevalence of pre-procedural TIMI flow grade 0 compared with those without IMH (82.9% vs 78.0%), with a significant trend difference in pre - interventional TIMI flow grades between the two groups (*P* = 0.022). The use of glycoprotein IIb/IIIa inhibitors was significantly more common in the IMH + group than in the IMH - group (87.3% vs 74.9%, *P* = 0.001). CMR imaging revealed more severe myocardial injury in the IMH+ group, characterized by larger infarct size (26.67% ± 10.76% vs 21.64% ± 10.35%, *P* < 0.001), lower LVEF (46.27% ± 11.29% vs 50.05% ± 10.97%, *P* < 0.001), and a higher incidence of MVO(68.8% vs. 53.3%, *P* = 0.001). In the SHR quartile analysis (**Supplementary Table S1**), patients with higher SHR levels exhibited a stepwise increase in IMH prevalence (ANOVA *P* < 0.001). The incidence of IMH increased progressively across SHR quartiles, ranging from 25.0% in the lowest quartile (Q1) to 56.2% in the highest quartile (Q4). This corresponds to an absolute risk difference of 31.2% between patients with the highest and lowest SHR levels. The Q4 group (SHR>1.01) demonstrated significantly larger infarct size (26.46% vs 21.67% in Q1) and lower LVEF (45.88% vs 50.61% in Q1). The proportion of patients with diabetes was also significantly higher in Q4 (45.5% vs Q1: 21.3%), with significantly elevated HbA1c levels(median 6.10% vs 5.90% in Q1) and FBG (median 8.36 mmol/L vs 4.99 mmol/L in Q1)(all *P* < 0.0083).

### 3.2 Association between fasting SHR and the presence of IMH

**Table 2**​​ presents the univariate and multivariable logistic regression analyses for predictors of IMH. In univariate analysis, elevated fasting SHR, peak cardiac troponin I levels, greater infarct size, lower LVEF, the presence of MVO, history of hypercholesterolemia, worse pre-procedural TIMI flow, and the use of glycoprotein IIb/IIIa inhibitors were associated with the presence of IMH. The Variance Inflation Factors for all covariates ranged from 1.03 to 2.09, indicating no significant multicollinearity among the included variables. Multivariate analysis identified fasting SHR as a robust independent predictor of IMH (adjusted OR: 1.21 per 0.1-unit increase, 95% CI: 1.10–1.33, *P* < 0.001, **Table 2**, Model1). This association was further confirmed by hierarchical regression analysis, which demonstrated that the predictive value of SHR remained consistent after sequential adjustment for demographics, clinical variables, biomarkers, and CMR parameters (ORs range: 1.21–1.23, all *P* < 0.001, **Supplementary Table S2**). Furthermore, the predictive performance of SHR was superior to that of FBG alone. When SHR was replaced by FBG, the association was markedly attenuated and did not reach statistical significance (adjusted OR: 1.06, 95% CI: 0.99–1.13, *P* = 0.124, **Table 2****, ​​**Model 2).

**Table 2:** Univariate and multivariable logistic regression analysis for predictors of IMH.

**Variable**	Univariable OR (95% CI)	*P* value	Multivariable Model 1 OR (95% CI)	*P* value	Multivariable Model 2 OR (95% CI)	*P* value
Female	0.62 (0.36–1.10)	​**​0.100​**​	0.53 (0.29 – 0.99)	​**​0.045**	0.62 (0.34–1.12)	0.113
Age, year	1.00 (0.98–1.02)	0.868	–	–	–	–
Body mass index, kg/m²	1.04 (0.98–1.10)	0.211	–	–	–	–
Hypertension	1.16 (0.81–1.66)	0.426	–	–	–	–
Diabetes mellitus	1.00 (0.67–1.49)	0.994	–	–	–	–
Hypercholesterolemia	1.57 (1.09–2.27)	​**​0.016​**​	1.28 (0.85 – 1.94)	0.233	1.32 (0.88–1.99)	0.182
Smoking	1.06 (0.74–1.51)	0.772	–	–	–	–
Fasting blood glucose, mmol/L	1.06 (1.00–1.13)	​**​0.065​**​	–	–	1.06 (0.99–1.13)	0.124
**HbA1c, %​​**	0.94 (0.83–1.07)	​**​0.359**	–	–	–	–
Fasting SHR, per 0.1 increase	1.21 (1.10–1.33)	​**​<0.001​**​	1.21 (1.10 – 1.33)	​**​<0.001**	–	–
Triglycerides, mmol/L	0.99 (0.94–1.03)	0.536	–	–	–	–
HDL cholesterol, mmol/L	0.68 (0.34–1.35)	0.267	–	–	–	–
LDL cholesterol, mmol/L	0.99 (0.96–1.02)	0.584	–	–	–	–
Creatinine, μmol/L	1.00 (0.99–1.01)	0.964	–	–	–	–
Peak cTnI, ng/mL	**1.01 (1.01–1.02)**	**0.001**	1.00 (1.00 – 1.01)	0.373	1.00 (1.00–1.01)	0.294
Anterior infarct	1.42 (0.98–2.06)	​**​0.062​**​	1.08 (0.71 – 1.64)	0.72	1.08 (0.72–1.63)	0.708
Killip class	1.51 (0.69–3.28)	0.299	–	–	–	–
Pre-TIMI flow	0.69 (0.51–0.93)	​**​0.017​**​	0.93 (0.65 – 1.32)	0.675	0.90 (0.64–1.28)	0.554
Post-TIMI flow	0.69 (0.33–1.44)	0.329	–	–	–	–
Reperfusion time	1.00 (1.00–1.00)	0.236	–	–	–	–
Aspirin	0.46 (0.13–1.66)	0.237	–	–	–	–
P2Y12 receptor inhibitor	0.83 (0.53–1.30)	0.423	–	–	–	–
**​GP IIb/IIIa Inhibitors​​**	2.31 (1.41–3.76)	**0.001**	2.42 (1.41 – 4.16)	​**​0.001​**​	2.34 (1.38–3.98)	​**​0.002**
​**​Beta-blockers​​**	1.11 (0.67–1.84)	0.685	–	–	–	–
**​ACE Inhibitors​​**	0.87 (0.59–1.27)	0.469	–	–	–	–
Infarct size, % LV	1.05 (1.03–1.07)	​**​<0.001​**​	1.03 (1.00 – 1.05)	​**​0.046**	1.03 (1.00–1.05)	**0.042**
LVEF, %	0.97 (0.95–0.99)	​**​<0.001​**​	0.99 (0.97–1.02)	0.495	0.99 (0.97–1.01)	0.351
MVO present	1.93 (1.33–2.81)	​**​0.001​**​	1.52 (0.99 – 2.33)	​**​0.053**	1.42 (0.93–2.16)	0.101

OR, odds ratio; CI, confidence interval. Other abbreviations as in **Table 1**.

Continuous variables are expressed as OR per 1-unit increase, except for fasting stress hyperglycemia ratio (SHR), which is expressed as OR per 0.1-unit increase.

Multivariable model included variables with *P* < 0.1 (in bold) in univariate analysis after excluding collinearity.

Model 1: Glycemic parameter included only fasting SHR.

Model 2: Glycemic parameters included only fasting blood glucose; fasting SHR was excluded due to collinearity with fasting blood glucose, and HbA1c was excluded due to non-significance in univariate analysis.

Causal mediation analysis revealed that infarct size partially mediated the association between SHR and IMH. The ACME of infarct size was 0.0025 (95% CI: 0.0006-0.0100, *P* = 0.004). However, the ADE of SHR on IMH remained highly significant (ADE: 0.0157, 95% CI: 0.0099-0.0198, *P* < 0.001). The proportion of the total effect mediated by infarct size was approximately 13.5% (95% CI: 4.4%-27.9%, *P* = 0.004), indicating that the majority of the impact of SHR on IMH is direct and independent of infarct size. In contrast, MVO did not show a significant mediation effect (ACME: -0.001, 95% CI: -0.003-0.000, *P* = 0.85), whereas the direct effect of SHR on IMH remained significant in this model (ADE: 0.018, 95% CI: 0.010-0.022, *P* < 0.001).

To assess whether the association between fasting SHR and IMH was consistent regardless of chronic glycemic status, we conducted stratified analyses based on the presence of diabetes mellitus (**Table 3**). Interaction analysis revealed no significant difference in the association between fasting SHR and IMH between the two groups (*P* for interaction=0.499). Consequently, fasting SHR remained a significant independent predictor of IMH in both non-diabetic (adjusted OR: 1.27 per 0.1-unit increase, 95% CI: 1.10–1.45; *P* = 0.001) and diabetic patients (adjusted OR: 1.21 per 0.1-unit increase, 95% CI: 1.04–1.42; *P* = 0.015).

**Table 3:** Association between fasting SHR and IMH stratified by diabetes status.

Variable	Non-diabetes (n=358)				Diabetes (n=138)			
	Unadjusted OR	*P* value	Adjusted OR	*P* value	Unadjusted OR	*P* value	Adjusted OR	*P* value
	(95% CI)		(95% CI)		(95% CI)		(95% CI)	
Female	0.65 (0.33–1.28)	0.215	–	–	0.56 (0.20–1.57)	0.271	–	–
Age, year	1.01 (0.99–1.03)	0.606	–	–	0.97 (0.94–1.01)	0.175	–	–
Body mass index, kg/m²	1.02 (0.95–1.09)	0.678	–	–	1.08 (0.98–1.20)	0.121	–	–
Hypertension	1.10 (0.72–1.67)	0.668	–	–	1.40 (0.66–2.96)	0.379	–	–
Hypercholesterolemia	1.42 (0.91–2.19)	0.121	–	–	2.07 (1.04–4.11)	**0.039**	1.46 (0.66–3.24)	0.353
Smoking	0.95 (0.62–1.45)	0.825	–	–	1.37 (0.69–2.73)	0.365	–	–
Fasting SHR, per 0.1 increase	1.28 (1.12-1.46)	​**​<0.001​**​	1.27 (1.10–1.45)	​**​0.001​**​	1.16 (1.01–1.33)	**0.036**	1.21 (1.04–1.42)	**0.015**
Triglycerides, mmol/L	1.04 (0.92–1.17)	0.533	–	–	0.97 (0.86–1.09)	0.578	–	–
HDL cholesterol, mmol/L	0.45 (0.20–1.02)	**0.057**	0.32 (0.13–0.78)	​**​0.012​**​	2.50 (0.56–11.13)	0.231	–	–
LDL cholesterol, mmol/L	0.99 (0.93–1.05)	0.684	–	–	1.35 (0.93–1.95)	0.114	–	–
Creatinine, μmol/L	1.00 (0.99–1.01)	0.779	–	–	1.00 (0.98–1.01)	0.752	–	–
Peak cTnI, ng/mL	1.01 (1.00-1.02)	**0.022**	1.00 (0.99–1.01)	0.885	1.02 (1.01-1.03)	**0.004**	1.02 (1.00–1.03)	**0.022**
Anterior infarct	1.24 (0.80–1.93)	0.33	–	–	1.99 (0.99–4.00)	**0.053**	1.85 (0.81–4.21)	0.145
Killip class	2.05 (0.74–5.71)	0.168	–	–	0.77 (0.17–3.46)	0.729	–	–
Pre-TIMI flow	0.65 (0.46–0.93)	​**​0.020​**​	0.93 (0.63–1.40)	0.739	0.81 (0.43–1.52)	0.51	–	–
Post-TIMI flow (per grade)	1.18 (0.42–3.33)	0.75	–	–	0.30 (0.06–1.43)	0.131	–	–
Reperfusion time, min	1.00 (1.00–1.00)	0.412	–	–	1.00 (1.00–1.00)	0.338	–	–
Aspirin	0.70 (0.14–3.52)	0.666	–	–	0.70 (0.14–3.52)	0.666	–	–
P2Y12 receptor inhibitor	0.88 (0.52–1.49)	0.637	–	–	0.88 (0.52–1.49)	0.637	–	–
GP IIb/IIIa inhibitors	2.20 (1.22–3.96)	​**​0.009​**​	2.45 (1.30–4.63)	​**​0.006​**​	2.20 (1.22–3.96)	**0.009**	2.65 (0.93–7.49))	0.067
Beta-blockers	1.13 (0.61–2.10)	0.695	–	–	1.13 (0.61–2.10)	0.695	–	–
ACE inhibitors	0.98 (0.62–1.53)	0.916	–	–	0.98 (0.62–1.53)	0.916	–	–
Infarct size, % LV	1.05 (1.03–1.07)	​**​<0.001​**​	1.03 (1.00–1.06)	​**​0.041​**​	1.05 (1.01–1.09)	**0.016**	0.99 (0.93–1.05)	0.706
LVEF, %	0.97 (0.95–0.99)	​**​0.002​**​	0.99 (0.97–1.02)	0.598	0.97 (0.94–1.00)	**0.07**	0.97 (0.93–1.02)	0.196
MVO present	1.90 (1.23–2.94)	​**​0.004​**​	1.59 (0.97–2.60)	0.066	2.10 (0.98–4.51)	**0.057**	2.64 (1.03–6.78)	**0.043**

OR, odds ratio; CI, confidence interval. Other abbreviations as in **Table 1**.

Continuous variables are expressed as OR per 1-unit increase, except for fasting stress hyperglycemia ratio (SHR), which is expressed as OR per 0.1-unit increase.

Multivariable model included variables with *P* < 0.1 (in bold) in univariate analysis after excluding collinearity.

### 3.3 Nonlinear dose-response relationship between fasting SHR and IMH risk

**Figure 3:**
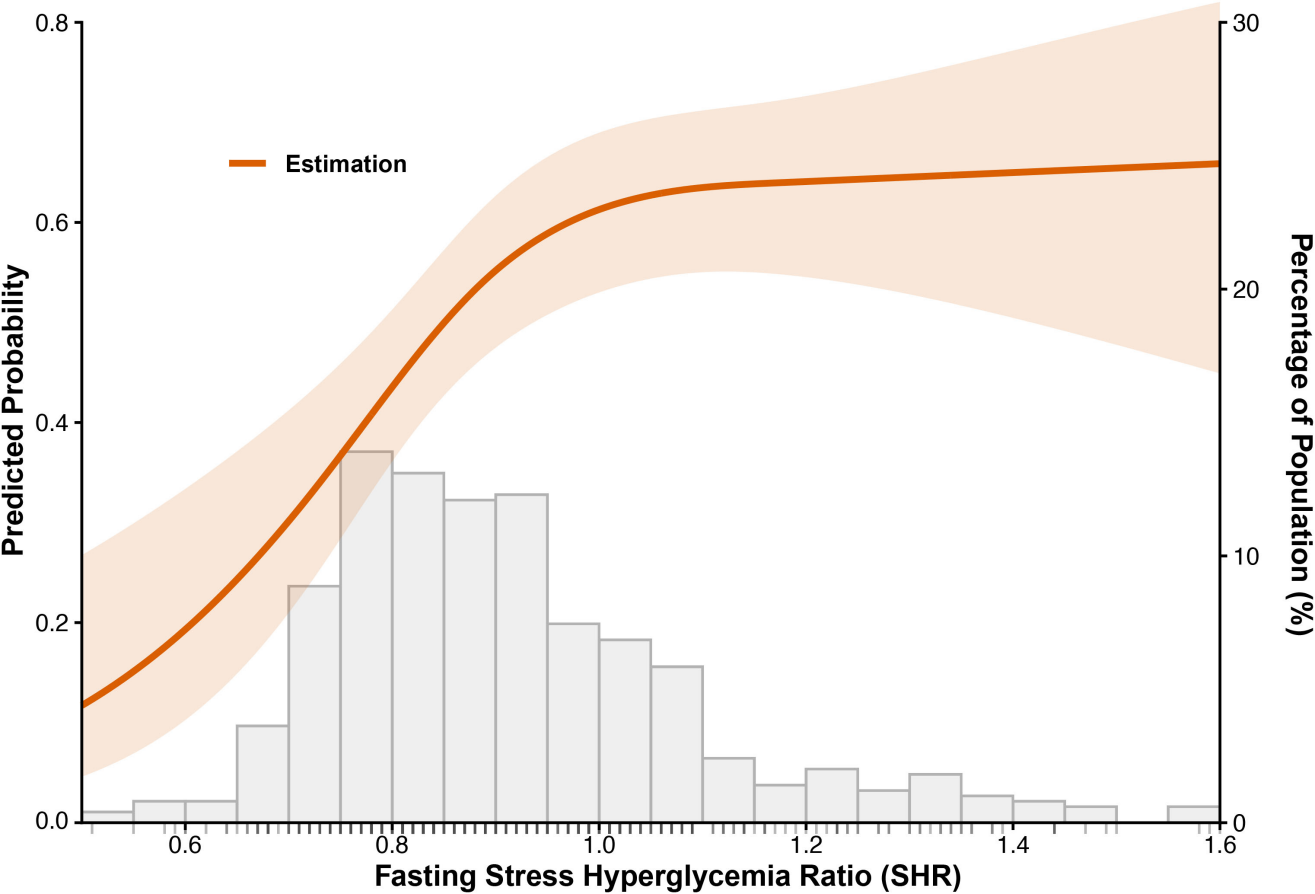
Nonlinear dose-response relationship between fasting Stress Hyperglycemia Ratio (SHR) and risk of Intramyocardial Hemorrhage (IMH). The solid orange line represents the point estimates of the predicted probabilities of IMH, with the surrounding light orange shaded band depicting the 95% confidence interval. The gray bars at the bottom represent the distribution of the study population (percentage) across different SHR levels, and the rug plot along the x-axis indicates individual data points. The model was adjusted for key clinical confounders, including infarct size (fixed at 23.9%), presence of microvascular obstruction (present), age (60 years), sex (male), and use of Glycoprotein IIb/IIIa inhibitors (yes).

RCS analysis revealed a significant nonlinear dose-response relationship between fasting SHR and the predicted probability of IMH (*P* for overall < 0.001, *P* for nonlinearity = 0.004; **Figure 3**). After adjustment for age, sex, infarct size, MVO, and glycoprotein IIb/IIIa inhibitor use, the fitted curve demonstrated a steep ascent in IMH risk at lower SHR levels, where the superimposed histogram indicated a high concentration of the study population. Specifically, the predicted probability of IMH rose from approximately 0.20 at an SHR level of 0.6 to over 0.60 at an SHR level of 1.0. Beyond an SHR of 1.0, the trajectory became more gradual; however, confidence intervals widened at these extremes, corresponding to a sparse distribution of patients as shown by the histogram.

### 3.4 Association between fasting SHR and clinical outcomes

**Figure 4:**
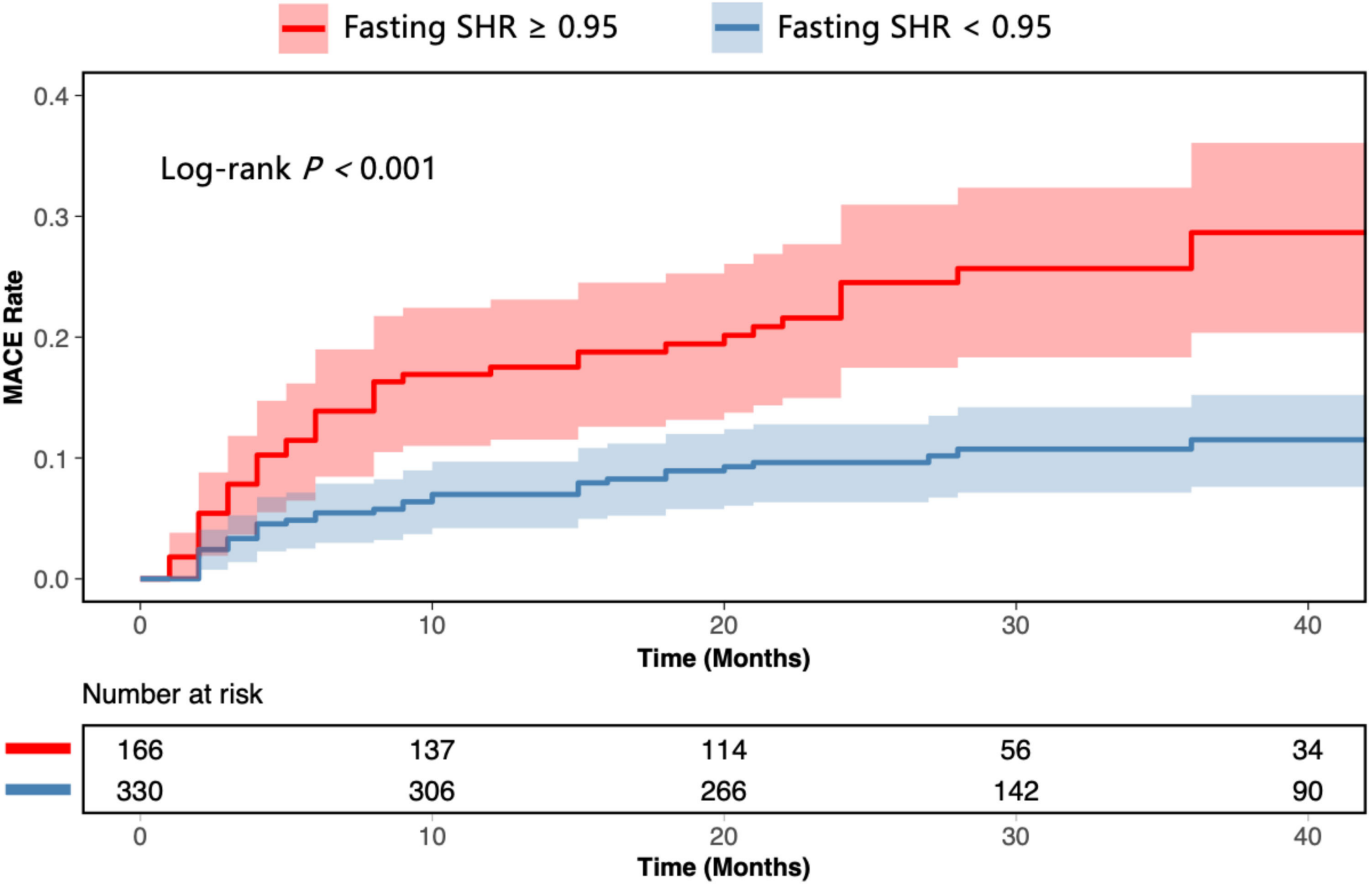
Kaplan-Meier curves for cumulative major adverse cardiac events (MACE) incidence. Patients were stratified into two groups based on an optimal fasting stress hyperglycemia ratio (SHR) cutoff value of 0.95, which was derived from maximally selected rank statistics. The solid lines represent the cumulative incidence rates, and the shaded areas represent the 95% confidence intervals.

During a median follow-up of 25 months (IQR: 22–40 months), MACE occurred in 76 (15%) participants of the 496 participants, with 15 individuals (3%) lost to follow-up. The composite endpoint consisted of 11 (2%) deaths, 22 (4%) cases of myocardial reinfarction, 39 (8%) cases of heart failure, and 4 (1%) strokes. In univariate Cox regression analysis, each 0.1-unit increment in fasting SHR was associated with a 20% increased risk of MACE (Unadjusted HR: 1.20; 95% CI: 1.11–1.31; *P* < 0.001). This association remained robust and statistically significant after adjustment for key clinical covariates including age, sex, hypertension, diabetes mellitus, Killip class, and anterior infarction location(Adjusted HR: 1.20; 95% CI: 1.09–1.31; *P* < 0.001). Notably, the inclusion of SHR significantly improved the model’s predictive performance beyond standard clinical risk factors. The Harrell’s C-index increased from 0.585 for the baseline model to 0.642 for the full model, representing a net improvement of 0.057 (*P* < 0.001). To address the heterogeneity of the composite endpoint, we further analyzed the association between SHR and individual MACE components. In the univariate analysis, each 0.1-unit increment in SHR was significantly associated with an increased risk of all-cause death (HR: 1.32; 95% CI: 1.08–1.61; *P* = 0.006) and heart failure (HR: 1.23; 95% CI: 1.10–1.38; *P* < 0.001). While a positive trend was observed for myocardial reinfarction, it did not reach statistical significance (HR: 1.13; 95% CI: 0.94–1.34; *P* = 0.189). No significant association was found for stroke (HR: 0.97; 95% CI: 0.58–1.62; *P* = 0.908), likely due to the low number of events (n=4). Correspondingly, Kaplan–Meier survival curves demonstrated a significantly higher cumulative incidence of MACE in patients with elevated SHR. Using the optimal cutoff value of 0.95 derived from maximally selected rank statistics, patients with SHR ≥ 0.95 had a markedly worse prognosis compared to those with fasting SHR < 0.95 (log-rank test *P* < 0.001; **Figure 4**). The worst-case scenario sensitivity analysis yielded consistent results, indicating an increased MACE risk with each 0.1-unit increment in SHR (unadjusted HR: 1.17; 95% CI: 1.08–1.28; *P* < 0.001; adjusted HR: 1.17; 95% CI: 1.07–1.28; *P* = 0.001).

## 4 Discussion

This study utilized data from a prospective, multicenter registry of consecutive patients with STEMI undergoing PPCI to examine the association between the fasting SHR and CMR-defined IMH as well as adverse clinical outcomes. Our principal findings were as follows: (1) Fasting SHR is a potent and independent predictor of IMH in reperfused STEMI, outperforming conventional glycemic parameters such as FBG and HbA1c. (2) A nonlinear dose-response relationship was identified, characterized by a rapid increase in IMH risk at lower SHR levels, where the study population was primarily concentrated, and remained consistently high thereafter. (3) Elevated fasting SHR was significantly associated with increased risk of MACE. These findings underscore the strong association of acute metabolic stress, rather than chronic dysglycemia, with severe microvascular injury following STEMI. Consequently, our study supports the integration of fasting SHR into early risk stratification to identify patients at high risk for IMH, who may benefit from intensified monitoring and novel therapies targeting acute glucose metabolism and microvascular protection.

The relationship between hyperglycemic markers and the development of IMH has remained less clearly defined in existing literature. To date, only one small single-center study (22) which included 28 patients with IMH, has suggested that admission hyperglycemia is an independent predictor of IMH. Although that study provided initial evidence supporting a glucose–IMH association, a key limitation of relying solely on admission blood glucose is its inability to differentiate acute stress-induced hyperglycemia from chronic hyperglycemia. To address this, the SHR was developed to provide a more accurate measure of “true” stress by adjusting glucose levels for baseline HbA1c (11). Extensive research has demonstrated that admission-based SHR is significantly associated with increased mortality in patients with acute myocardial infarction, serving as a robust prognostic marker independent of diabetes status (23, 24). Despite its clinical utility, admission SHR remains highly susceptible to the “last meal effect”, including the timing and carbohydrate composition of pre-hospital intake. As a non-fasting measure, admission SHR introduces considerable random error and lacks the rigorous standardization required for reproducible clinical assessment. This variability is further compounded by the physiological nature of the acute glycemic response. While admission SHR often reflects a transient “sympathetic storm” triggered by acute pain or the immediate physiological shock of the event, fasting SHR captures the patient’s sustained metabolic derangement and underlying insulin resistance (12, 13). Crucially, this standardized approach ensures that the glycemic assessment remains a robust reflection of metabolic status, as it is less influenced by early emergency glycemic interventions administered during the hyper-acute phase. Consequently, by employing fasting SHR, our study provides a more stable and physiologically grounded assessment of how stress-induced hyperglycemia relates to the development of IMH.

The association between elevated fasting SHR and IMH may be mediated through interconnected pathways involving endothelial dysfunction, oxidative stress, and microvascular disruption (7, 25). Acute hyperglycemia, reflected by high SHR, exacerbates oxidative stress and reactive oxygen species production, damaging endothelial tight junctions and increasing vascular permeability (13), while simultaneously upregulating matrix metalloproteinase-9, which degrades the extracellular matrix and weakens capillary integrity (22). Additionally, elevated SHR levels may exacerbate the inflammatory milieu through elevated pro-inflammatory cytokines, promoting neutrophil infiltration, neutrophil extracellular trap formation, and further microvascular injury (13). Crucially, this metabolic susceptibility appears distinct from thrombotic complications. The association between SHR and IMH remained robust after adjusting for GP IIb/IIIa inhibitor use—a surrogate for high procedural thrombotic burden—suggesting that the risk conferred by stress hyperglycemia is driven by the direct toxic effects described above, rather than being confounded by baseline thrombus load.

Furthermore, our interaction analysis demonstrated that the association between elevated SHR and IMH risk is consistent across both diabetic and non-diabetic populations (*P* for interaction = 0.499). This implies that the mechanisms of acute glycemic injury—such as oxidative stress and endothelial dysfunction—may operate independently of chronic metabolic adaptations (26). Our findings highlight the need for vigilance in non-diabetic patients, a subgroup traditionally overlooked for hyperglycemia-related complications, yet who remain just as vulnerable to the adverse effects of acute stress hyperglycemia.

The nonlinear dose-response relationship provides further insights into the impact of stress hyperglycemia. Our RCS analysis revealed a rapid increase in IMH risk at lower SHR levels, where the study population was primarily concentrated, and remained consistently high thereafter. This distribution highlights the sensitivity of fasting SHR as a predictive marker for IMH and indicates that the observed risk trajectory is clinically relevant to a broad patient demographic rather than being driven by outliers. These findings suggest that early management of stress hyperglycemia—initiated before SHR reaches markedly elevated levels—might be effective in mitigating the risk of IMH.

### 4.1 Limitations

Several limitations of our study should be acknowledged. First, due to the study’s observational design, we cannot establish causality. The observed association may be subject to reverse causality, as a greater extent of myocardial injury and microvascular dysfunction could potentially exacerbate systemic stress and subsequently elevate SHR values. However, in hierarchical regression models, the relationship between SHR and IMH remained robust (ORs: 1.21–1.23, all *P* < 0.001) even after comprehensive adjustment for peak cardiac troponin I and CMR-derived severity markers (infarct size, LVEF, and MVO). Furthermore, our causal mediation analysis indicated that infarct size mediated only a minor fraction (13.5%) of this relationship, with a predominant direct effect (ADE: 0.0157, *P* < 0.001), and MVO showed no significant mediation. Collectively, these findings support fasting SHR as an independent predictor of severe microvascular injury, rather than merely serving as a passive surrogate for infarct burden. Second, despite our use of a prospective, multicenter registry and comprehensive multivariable adjustments, the potential for residual confounding due to unmeasured or unobserved factors cannot be entirely excluded. For instance, unmeasured inflammatory markers (such as high-sensitivity C-reactive protein, interleukin-6, or neutrophil count), stress hormones (like catecholamines and cortisol), insulin use, and peri-procedural glucose control could potentially influence both SHR and IMH development. While we adjusted for a wide array of clinically relevant variables, the existence of such unmeasured confounders might still subtly bias the observed associations and could affect the generalizability of our findings to populations with different unmeasured characteristics. Third, we acknowledge the gender imbalance in our study cohort, where approximately 90% of the patients were male. While this predominance reflects the typical demographic of STEMI populations in our region, it may limit the generalizability of our findings to female patients. Given that recent evidence has highlighted significant sex-related differences in clinical presentation, pathophysiology, and outcomes following acute myocardial infarction (27), future studies with larger, more balanced cohorts are warranted to validate our findings in the female population. Finally, while our study indicated a significant association between elevated fasting SHR and adverse clinical outcomes, its translation into routine clinical practice requires validation in broader, multi-center STEMI cohorts. Future studies should establish disease-specific SHR thresholds optimized for clinical decision-making and evaluate the effectiveness of SHR-guided interventions in improving patient outcomes.

## 5 Conclusions

Fasting SHR is a potent independent predictor of IMH in patients with STEMI following PPCI. Notably, the IMH risk escalates rapidly even at lower SHR levels, underscoring the critical need for early management of stress hyperglycemia. Elevated fasting SHR is significantly associated with an increased risk of MACE. These findings establish fasting SHR not only as a biomarker for microvascular injury but also as a pivotal tool for early risk stratification in STEMI.

## Data Availability

The raw data supporting the conclusions of this article will be made available by the authors, without undue reservation.

## References

[1] Tsao CW Aday AW Almarzooq ZI Alonso A Beaton AZ Bittencourt MS . Heart disease and stroke statistics-2022 update: A report from the american heart association. Circulation. (2022) 145:e153–639. doi: 10.1161/CIR.0000000000001052, PMID: 35078371

[2] Lechner I Reindl M Stiermaier T Tiller C Holzknecht M Oberhollenzer F . Clinical outcomes associated with various microvascular injury patterns identified by cmr after stemi. J Am Coll Cardiol. (2024) 83:2052–62. doi: 10.1016/j.jacc.2024.03.408, PMID: 38777509

[3] Vora KP Kalra A Shah CD Bhatt K Kumar A Pandya T . In-hospital mortality in hemorrhagic myocardial infarction. NEJM Evid. (2025) 4:EVIDoa2400294. doi: 10.1056/EVIDoa2400294, PMID: 40858097

[4] Kumar A Connelly K Vora K Bainey KR Howarth A Leipsic J . The canadian cardiovascular society classification of acute atherothrombotic myocardial infarction based on stages of tissue injury severity: an expert consensus statement. Can J Cardiol. (2024) 40:1–14. doi: 10.1016/j.cjca.2023.09.020, PMID: 37906238 PMC13016900

[5] Vora KP Kumar A Krishnam MS Prato FS Raman SV Dharmakumar R . Microvascular obstruction and intramyocardial hemorrhage in reperfused myocardial infarctions: pathophysiology and clinical insights from imaging. JACC Cardiovasc Imaging. (2024) 17:795–810. doi: 10.1016/j.jcmg.2024.02.003, PMID: 38613553 PMC13159459

[6] Bergamaschi L Landi A Maurizi N Pizzi C Leo LA Arangalage D . Acute response of the noninfarcted myocardium and surrounding tissue assessed by T2 mapping after stemi. JACC Cardiovasc Imaging. (2024) 17:610–21. doi: 10.1016/j.jcmg.2023.11.014, PMID: 38276932

[7] Zavadovsky KV Ryabov VV Vyshlov EV Mochula OV Sirotina M Kan A . Intra-myocardial hemorrhage and cardiac microvascular injury in ischemia/reperfusion. A systematic review of current evidences. Curr Probl Cardiol. (2025) 50:102918. doi: 10.1016/j.cpcardiol.2024.102918, PMID: 39510400

[8] Amier RP Tijssen RYG Teunissen PFA Fernandez-Jimenez R Pizarro G Garcia-Lunar I . Predictors of intramyocardial hemorrhage after reperfused st-segment elevation myocardial infarction. J Am Heart Assoc. (2017) 6:e005651. doi: 10.1161/JAHA.117.005651, PMID: 28862937 PMC5586425

[9] Tiller C Reindl M Holzknecht M Lechner I Oberhollenzer F von der Emde S . Association of intramyocardial hemorrhage with inflammatory biomarkers in patients with st-segment elevation myocardial infarction. JACC Adv. (2025) 4:101647. doi: 10.1016/j.jacadv.2025.101647, PMID: 40080922 PMC11953969

[10] Capes SE Hunt D Malmberg K Gerstein HC . Stress Hyperglycaemia and Increased Risk of Death after Myocardial Infarction in Patients with and without Diabetes: A Systematic Overview. Lancet. (2000) 355:773–8. doi: 10.1016/S0140-6736(99)08415-9, PMID: 10711923

[11] Roberts GW Quinn SJ Valentine N Alhawassi T O'Dea H Stranks SN . Relative hyperglycemia, a marker of critical illness: introducing the stress hyperglycemia ratio. J Clin Endocrinol Metab. (2015) 100:4490–7. doi: 10.1210/jc.2015-2660, PMID: 26485219

[12] Cui K Fu R Yang J Xu H Yin D Song W . The Impact of Fasting Stress Hyperglycemia Ratio, Fasting Plasma Glucose and Hemoglobin A1c on in-Hospital Mortality in Patients with and without Diabetes: Findings from the China Acute Myocardial Infarction Registry. Cardiovasc Diabetol. (2023) 22:165. doi: 10.1186/s12933-023-01868-7, PMID: 37403082 PMC10320917

[13] Bo K Li W Zhang H Wang Y Zhou Z Gao Y . Association of stress hyperglycemia ratio with left ventricular function and microvascular obstruction in patients with st-segment elevation myocardial infarction: A 3.0 T cardiac magnetic resonance study. Cardiovasc Diabetol. (2024) 23:179. doi: 10.1186/s12933-024-02271-6, PMID: 38802898 PMC11131267

[14] Leng S Ge H He J Kong L Yang Y Yan F . Long-term prognostic value of cardiac mri left atrial strain in st-segment elevation myocardial infarction. Radiology. (2020) 296:299–309. doi: 10.1148/radiol.2020200176, PMID: 32544032

[15] Zhao Y Lu X Wan F Gao L Lin N He J . Disruption of circadian rhythms by shift work exacerbates reperfusion injury in myocardial infarction. J Am Coll Cardiol. (2022) 79:2097–115. doi: 10.1016/j.jacc.2022.03.370, PMID: 35618347 PMC8972444

[16] Pu J Ding S Ge H Han Y Guo J Lin R . Efficacy and safety of a pharmaco-invasive strategy with half-dose alteplase versus primary angioplasty in st-segment-elevation myocardial infarction: early-myo trial (Early routine catheterization after alteplase fibrinolysis versus primary pci in acute st-segment-elevation myocardial infarction). Circulation. (2017) 136:1462–73. doi: 10.1161/CIRCULATIONAHA.117.030582, PMID: 28844990

[17] Lai W Chen-Xu Z Jian-Xun D Jie H Ling-Cong K Dong-Ao-Lei A . Prognostic implications of left ventricular torsion measured by feature-tracking cardiac magnetic resonance in patients with st-elevation myocardial infarction. Eur Heart J Cardiovasc Imaging. (2022). doi: 10.1093/ehjci/jeac177, PMID: 36056877

[18] Alpert JS Thygesen K Antman E Bassand JP . Myocardial infarction redefined–a consensus document of the joint european society of cardiology/american college of cardiology committee for the redefinition of myocardial infarction. Eur Heart J. (2000) 21:1502–13. doi: 10.1053/euhj.2000.2305, PMID: 10987628

[19] O'Gara PT Kushner FG Ascheim DD Casey DE Jr. Chung MK de Lemos JA . 2013 Accf/Aha guideline for the management of st-Elevation myocardial infarction: A report of the american college of cardiology foundation/American heart association task force on practice guidelines. J Am Coll Cardiol. (2013) 61:e78–e140. doi: 10.1016/j.jacc.2012.11.019, PMID: 23256914

[20] O'Gara PT Kushner FG Ascheim DD Casey DE Jr. Chung MK de Lemos JA . 2013 Accf/Aha guideline for the management of st-Elevation myocardial infarction: A report of the american college of cardiology foundation/American heart association task force on practice guidelines. Circulation. (2013) 127:e362–425. doi: 10.1161/CIR.0b013e3182742cf6, PMID: 23247304

[21] Akl EA Kahale LA Agoritsas T Brignardello-Petersen R Busse JW Carrasco-Labra A . Handling trial participants with missing outcome data when conducting a meta-analysis: A systematic survey of proposed approaches. Syst Rev. (2015) 4:98. doi: 10.1186/s13643-015-0083-6, PMID: 26202162 PMC4511978

[22] Ota S Nishiguchi T Taruya A Tanimoto T Ino Y Katayama Y . Hyperglycemia and intramyocardial hemorrhage in patients with st-segment elevation myocardial infarction. J Cardiol. (2022) 80:456–61. doi: 10.1016/j.jjcc.2022.06.003, PMID: 35750553

[23] Marenzi G Cosentino N Milazzo V De Metrio M Cecere M Mosca S . Prognostic value of the acute-to-chronic glycemic ratio at admission in acute myocardial infarction: A prospective study. Diabetes Care. (2018) 41:847–53. doi: 10.2337/dc17-1732, PMID: 29382659

[24] Cui K Fu R Yang J Xu H Yin D Song W . Stress Hyperglycemia Ratio and Long-Term Mortality after Acute Myocardial Infarction in Patients with and without Diabetes: A Prospective, Nationwide, and Multicentre Registry. Diabetes Metab Res Rev. (2022) 38:e3562. doi: 10.1002/dmrr.3562, PMID: 35772392

[25] Zhang F Li Z Wang Y Li C Lu C . Mitochondrial dysfunction as a therapeutic target in diabetic cardiomyopathy: progress and prospects. CVIA. (2025) 10. doi: 10.15212/cvia.2024.0063

[26] Kolluru GK Bir SC Kevil CG . Endothelial dysfunction and diabetes: effects on angiogenesis, vascular remodeling, and wound healing. Int J Vasc Med. (2012) 2012:918267. doi: 10.1155/2012/918267, PMID: 22611498 PMC3348526

[27] Canton L Fedele D Bergamaschi L Foa A Di Iuorio O Tattilo FP . Sex- and age-related differences in outcomes of patients with acute myocardial infarction: minoca vs. Mioca. Eur Heart J Acute Cardiovasc Care. (2023) 12:604–14. doi: 10.1093/ehjacc/zuad059, PMID: 37261384

