# Supplementary material for: Fasting stress hyperglycemia ratio as a predictor of intramyocardial hemorrhage and adverse outcomes in ST-segment elevation myocardial infarction
Source: Front Endocrinol (Lausanne). 2026 Feb 11;17:1761471. doi: 10.3389/fendo.2026.1761471 (PMC12932205; doi:10.3389/fendo.2026.1761471)
Supplement: Supplementary file 1 [file Table1.docx]

**Supplementary Table S1 Baseline Characteristics of the Study Population Stratified by fasting Stress Hyperglycemia Ratio (SHR) Quartiles​​**

| **Variable​**​ | **Q1 (n=136)​​**  **SHR ≤ 0.79** | **​​Q2 (n=116)**  **0.79 < SHR ≤ 0.89​​** | **​​Q3 (n=123)​​**  **0.89 < SHR ≤ 1.01** | **​​Q4 (n=121)​​**  **SHR > 1.01** | ***P* Value** |
| --- | --- | --- | --- | --- | --- |
| **Demographic and Clinical Characteristics​​** | | | | | |
| Age, years | 58.13±9.95 | 58.08±10.75 | 58.03±10.65 | 59.47±9.11 | 0.631 |
| Male sex, n (%) | 125 (91.9) | 101 (87.1) | 108 (87.8) | 99 (81.8) | 0.115 |
| Body mass index**, kg/m²** | 24.77±3.26 | 24.99±2.71 | 25.02±2.95 | 25.46±3.59 | 0.370 |
| SBP, mmHg | 131.49±21.01 | 126.68±19.89 | 127.23±20.52 | 127.79±22.97 | 0.246 |
| DBP, mmHg | 80.49±11.80 | 77.44±12.13 | 78.90±14.36 | 77.42±14.16 | 0.192 |
| **Medical History** |  |  |  |  |  |
| Hypertension, n (%) | 74 (54.4) | 61 (52.6) | 67 (54.5) | 73 (60.3) | 0.646 |
| Diabetes, n (%) | 29 (21.3) | 23 (19.8) | 31 (25.2) | 55 (45.5)^abc^ | ​**​<0.001​** |
| Hyperlipidemia, n (%) | 47 (34.6) | 40 (34.5) | 44 (35.8) | 58 (47.9) | 0.085 |
| Current smoking, n (%) | 87 (64.0) | 64 (55.2) | 65 (52.8) | 56 (46.3) | ​**​0.039​** |
| **Laboratory Tests​**​ |  |  |  |  |  |
| **​​**Fasting blood glucose, mmol/L | 4.99 (4.59–5.96) | 5.58 (5.14–6.46)a | 6.00 (5.53–6.79)^ab^ | 8.36 (7.09–10.95)^abc^ | **<0.001** |
| **HbA1c, %** | 5.90 (5.60–6.60) | 5.80 (5.50–6.50) | 5.60 (5.30–6.23)^a^ | 6.10 (5.43–7.18)^c^ | **0.001** |
| Triglycerides, mmol/L | 1.48 (1.06–2.33) | 1.63 (1.18–2.42) | 1.61 (1.11–2.57) | 1.58 (1.14–2.40) | 0.799 |
| HDL cholesterol, mmol/L | 1.03 (0.88–1.22) | 1.04 (0.87–1.17) | 1.01 (0.86–1.22) | 1.03 (0.86–1.20) | 0.973 |
| LDL cholesterol, mmol/L | 3.14 (2.41–3.68) | 3.01 (2.46–3.76) | 3.15 (2.55–3.73) | 3.13 (2.56–3.62) | 0.933 |
| **​​** Creatinine, μmol/L | 76.95 (68.00–89.00) | 73.15 (64.55–86.50) | 77.00 (65.65–88.50) | 77.00 (65.00–87.00) | 0.518 |
| Peak cTnI, ng/mL | 23.10 (8.37 - 45.46) | 23.30 (7.91 - 42.25) | 27.86 (10.64 - 52.75) | 28.11 (13.20 - 59.50) | **0.029** |
| **Infarct Characteristics​**​ |  |  |  |  |  |
| Anterior infarct, n (%) | 77 (56.6) | 76 (65.5) | 68 (58.1) | 74 (61.2) | 0.454 |
| Killip class ≥2, n (%) | 6 (4.4) | 2 (1.7) | 4 (3.3) | 5 (4.1) | 0.812 |
| Preinterventional TIMI flow grade, n (%) |  |  |  |  |  |
| **0-1** | 123 (90.4) | 106 (91.4) | 113 (91.9) | 117 (96.7) | 0.241 |
| **2-3** | 13 (9.6) | 10 (8.6) | 10 (8.1) | 4 (3.3) |  |
| Postinterventional TIMI flow grade, n (%) |  |  |  |  |  |
| **3** | 131 (96.3) | 109 (94.0) | 120 (97.6) | 113 (93.4) | 0.361 |
| **0-2​​** | 5 (3.7) | 7 (6.0) | 3 (2.4) | 8 (6.6) |  |
| Onset to reperfusion, min | 270 (180–359) | 298.5 (206–430.5) | 285 (207.5–382.5) | 293 (204–415) | 0.263 |
| **Medications** |  |  |  |  |  |
| Aspirin | 134 (98.5) | 112 (96.6) | 122 (99.2) | 118 (97.5) | 0.486 |
| P2Y12 receptor inhibitor |  |  |  |  | 0.415 |
| Clopidogrel | 102 (75.0) | 97 (83.6) | 98 (79.7) | 96 (79.3) |  |
| Ticagrelor | 34 (25.0) | 19 (16.4) | 25 (20.3) | 25 (20.7) |  |
| **​GP IIb/IIIa Inhibitors​​** | 102 (75.0) | 94 (81.0) | 108 (87.8) | 93 (76.9) | 0.054 |
| ​**​Beta-blockers​​** | 111 (81.6) | 103 (88.8) | 103 (83.7) | 105 (86.8) | 0.393 |
| **​ACE Inhibitors​​** | 88 (64.7) | 80 (69.0) | 91 (74.0) | 79 (65.3) | 0.367 |
| **CMR Parameters​**​ |  |  |  |  |  |
| Onset to CMR, d | 4 (3-6) | 4.5 (3-6) | 5 (3-6) | 5 (3-6) | 0.132 |
| Infarct size, % LV | 21.67±10.20 | 24.10±11.31 | 22.95±10.97 | 26.46±10.26^a^ | ​**​0.004** |
| LVEF, % | 50.61±10.56 | 48.10±10.99 | 49.05±12.05 | 45.88±10.98^a^ | ​**​0.008​**​ |
| **MVO present​​** | 74 (54.4) | 70 (60.3) | 79 (64.2) | 73 (60.3) | 0.446 |
| **IMH present** | 34 (25.0) | 44 (37.9) | 59 (48.0)^a^ | 68 (56.2)^ab^ | **​<0.001​**​ |

**OR, odds ratio; CI, confidence interval. Other abbreviations as in Table 1.**

Values are mean ± SD, n (%), or median (interquartile range).

*P* values < 0.05 indicate significance (bolded).

^a^ *P* < 0.0083 versus Q1 (SHR ≤ 0.79)

^b^ *P* < 0.0083 versus Q2 (0.79 < SHR ≤ 0.89)

^c^ *P* < 0.0083 versus Q3 (0.89 < SHR ≤ 1.01)
