# Supplementary material for: Fasting stress hyperglycemia ratio as a predictor of intramyocardial hemorrhage and adverse outcomes in ST-segment elevation myocardial infarction
Source: Front Endocrinol (Lausanne). 2026 Feb 11;17:1761471. doi: 10.3389/fendo.2026.1761471 (PMC12932205; doi:10.3389/fendo.2026.1761471)
Supplement: Supplementary file 2 [file Table2.docx]

**Supplementary Table S2. Hierarchical logistic regression analysis for the association between SHR and IMH.**

|  | **Model 1** |  | **Model 2** |  | **Model 3** |  | **Model 4** |  |
| --- | --- | --- | --- | --- | --- | --- | --- | --- |
| **Variables** | **OR (95% CI)** | **P Value** | **OR (95% CI)** | **P Value** | **OR (95% CI)** | **P Value** | **OR (95% CI)** | **P Value** |
| Fasting SHR, per 0.1 increase | **1.23 (1.12–1.35)** | **<0.001** | **1.23 (1.12–1.36)** | **<0.001** | **1.22 (1.11–1.34)** | **<0.001** | **1.21 (1.10–1.33)** | **<0.001** |
| **Demographics** |  |  |  |  |  |  |  |  |
| Female | **0.50 (0.27–0.90)** | **0.021** | **0.52 (0.28–0.95)** | **0.034** | **0.52 (0.28–0.95)** | **0.033** | **0.53 (0.29–0.99)** | **0.045** |
| Hypercholesterolemia | **1.51 (1.04–2.20)** | **0.032** | 1.38 (0.94–2.05) | 0.105 | 1.34 (0.90–1.99) | 0.149 | 1.28 (0.85–1.94) | 0.233 |
| **Clinical Variables** |  |  |  |  |  |  |  |  |
| Anterior Infarct | - | - | 1.31 (0.89–1.94) | 0.173 | 1.29 (0.87–1.91) | 0.209 | 1.08 (0.71–1.64) | 0.720 |
| Pre-TIMI Flow | - | - | 0.73 (0.53–1.01) | 0.054 | 0.78 (0.56–1.08) | 0.128 | 0.93 (0.65–1.32) | 0.675 |
| GP IIb/IIIa inhibitors | - | - | **2.12 (1.26–3.56)** | **0.005** | **2.12 (1.26–3.59)** | **0.005** | **2.42 (1.41–4.16)** | **0.001** |
| **Biomarkers** |  |  |  |  |  |  |  |  |
| Peak cTnI, ng/mL | - | - | - | - | **1.01 (1.00–1.02)** | **0.011** | 1.00 (1.00–1.01) | 0.373 |
| **CMR Parameters** |  |  |  |  |  |  |  |  |
| Infarct Size, % LV | - | - | - | - | - | - | **1.03 (1.00–1.05)** | **0.046** |
| LVEF,% | - | - | - | - | - | - | 0.99 (0.97–1.02) | 0.495 |
| MVO Present | - | - | - | - | - | - | 1.52 (0.99–2.33) | 0.053 |

**OR, odds ratio; CI, confidence interval. Other abbreviations as in Table 1.**

Continuous variables are expressed as OR per 1-unit increase, except for fasting stress hyperglycemia ratio (SHR), which is expressed as OR per 0.1-unit increase.

*P* values < 0.05 indicate significance (bolded).
